# Susceptibility-Weighted Imaging Provides Insight into White Matter Damage in Amyotrophic Lateral Sclerosis

**DOI:** 10.1371/journal.pone.0131114

**Published:** 2015-06-25

**Authors:** Tino Prell, Viktor Hartung, Florian Tietz, Susanne Penzlin, Benjamin Ilse, Ferdinand Schweser, Andreas Deistung, Martin Bokemeyer, Jürgen R. Reichenbach, Otto W. Witte, Julian Grosskreutz

**Affiliations:** 1 Hans-Berger Department of Neurology, Jena University Hospital, Erlanger Allee 101, 07747, Jena, Germany; 2 Buffalo Neuroimaging Analysis Center, Department of Neurology, School of Medicine and Biomedical Sciences, State University of New York at Buffalo, New York, United States of America; 3 MRI Clinical and Translational Research Center, School of Medicine and Biomedical Sciences, State University of New York at Buffalo, New York, United States of America; 4 Medical Physics Group, Institute of Diagnostic and Interventional Radiology, Jena University Hospital, Philosophenweg 3, 07743, Jena, Germany; 5 Department of Neuroradiology, Jena University Hospital, Erlanger Allee 101, 07747, Jena, Germany; 6 Department of Radiology, HELIOS Kreiskrankenhaus Gotha, 99867, Gotha, Germany; 7 Department of Neurology, University of Göttingen, 37075, Göttingen, Germany; University of Ulm, GERMANY

## Abstract

**Background:**

Amyotrophic lateral sclerosis (ALS) is a fatal, progressive neurodegenerative disorder, characterised by widespread white matter damage. There is growing evidence that disturbances in iron metabolism contribute to white matter alterations.

**Materials & Methods:**

We analysed the data of susceptibility-weighted imaging (SWI) of white matter in a cohort of 27 patients with ALS and 30 healthy age-matched controls.

**Results:**

Signal alterations were found on SWI in the corpus callosum; along the corticospinal tract (subcortical motor cortex, posterior limb of the internal capsule and brainstem levels) and in the subgyral regions of frontal, parietal, temporal, occipital and limbic lobes. Alterations of white matter in the corpus callosum correlated with disease severity as assessed by the revised ALS functional rating scale.

**Conclusion:**

SWI is capable of indicating iron and myelin disturbances in white matter of ALS patients. The SWI patterns observed in this study suggest that widespread alterations due to iron disturbances occur in patients with ALS and correlate with disease severity.

## Introduction

Amyotrophic lateral sclerosis (ALS) is the most common adult-onset motor neuron disease and is characterised by progressive failure of the upper and lower motor neurons. Because the disease is not curable, patients usually die within 3 years due to respiratory failure. Understanding the underlying pathophysiology is crucial for the development of novel and effective therapeutic interventions. Several disease mechanisms are involved, including protein aggregation, increases in reactive oxygen species levels, calcium disturbances, mitochondrial degeneration and neuroinflammatory processes [1]. Similar to other neurodegenerative disorders like Parkinson or Alzheimer´s disease, magnetic resonance imaging (MRI) markers, indicative of excess iron levels, have been reported in the brains of patients with ALS as well as in animal models of ALS [2–5]. Abnormal iron or calcium levels can cause inflammation, microglia activation and oxidative damage in brain tissue. Since extensive white matter alterations are frequently observed in patients with ALS [6, 7], it seems reasonable to hypothesise a link between white matter damage and disturbances in iron levels.

We employed a statistical analysis of susceptibility-weighted imaging (SWI) data to assess patients with ALS. SWI involves a specific combination of phase and magnitude images from a T_2_*-weighted, high resolution gradient-echo sequence to create images with enhanced contrast that is exquisitely sensitive to compounds which distort the local magnetic field and as such make it useful in detecting venous blood, blood products, changes in iron storage, myelin or calcium [3, 8–10]. In the present study, we aimed to investigate whether SWI is able to identify particular characteristics of white matter alterations in patients with ALS. If successful, this result would provide further evidence for the role of disturbed iron levels and microstructural tissue damage in this neurodegenerative disorder.

## Methods

### Subject group

30 ALS patients (15 female and 15 male) from the Jena University Hospital (Jena, Germany) and 30 age- and sex-matched healthy controls (*P*_age_ = 0.18, student´s t-test) were enrolled into the study between 2011 and 2012. Three datasets of patients had to be excluded due to technical reasons. Therefore, MRI data from 27 ALS patients were used for the following analyses. ALS was diagnosed by two neurologists experienced in ALS (JG, TP). All patients had probable or definite ALS according to the revised El Escorial World Federation of Neurology diagnostic criteria. Mean age and mean disease duration were 63 (± 10) years and 21 (± 17) months, respectively. Neither age (*P* = 0.26) nor disease duration (*P* = 0.74) were significantly different between the genders (15 female, 12 male).

The patient group included 10 bulbar onsets and 17 spinal onsets. All patients were receiving riluzole and none were receiving psychoactive drugs. Disability was assessed using the revised ALS functional rating scale (ALSFRS-R) [11]. The mean ALSFRS-R was 38 points (standard deviation 6). Significant frontal or cognitive dysfunction was not observed in the ALS patients, although extensive neuropsychological assessment was not performed.

Control subjects (15 male, 15 female) had no history of central nervous system disease. Their neurological examinations were normal, and conventional T_1_- and T_2_-weighted MR images revealed no pathological findings. The mean age of the control group was 60 (± 8) years with no significant difference in age (*P* = 0.51) between genders. All patients submitted written informed consent and the study was approved by the local ethics committee (Ethik-Kommission der Friedrich-Schiller-Universität Jena an der Medizinischen Fakultät. Number: 3633-12/12).

### Data acquisition and preprocessing

**Fig 1:**
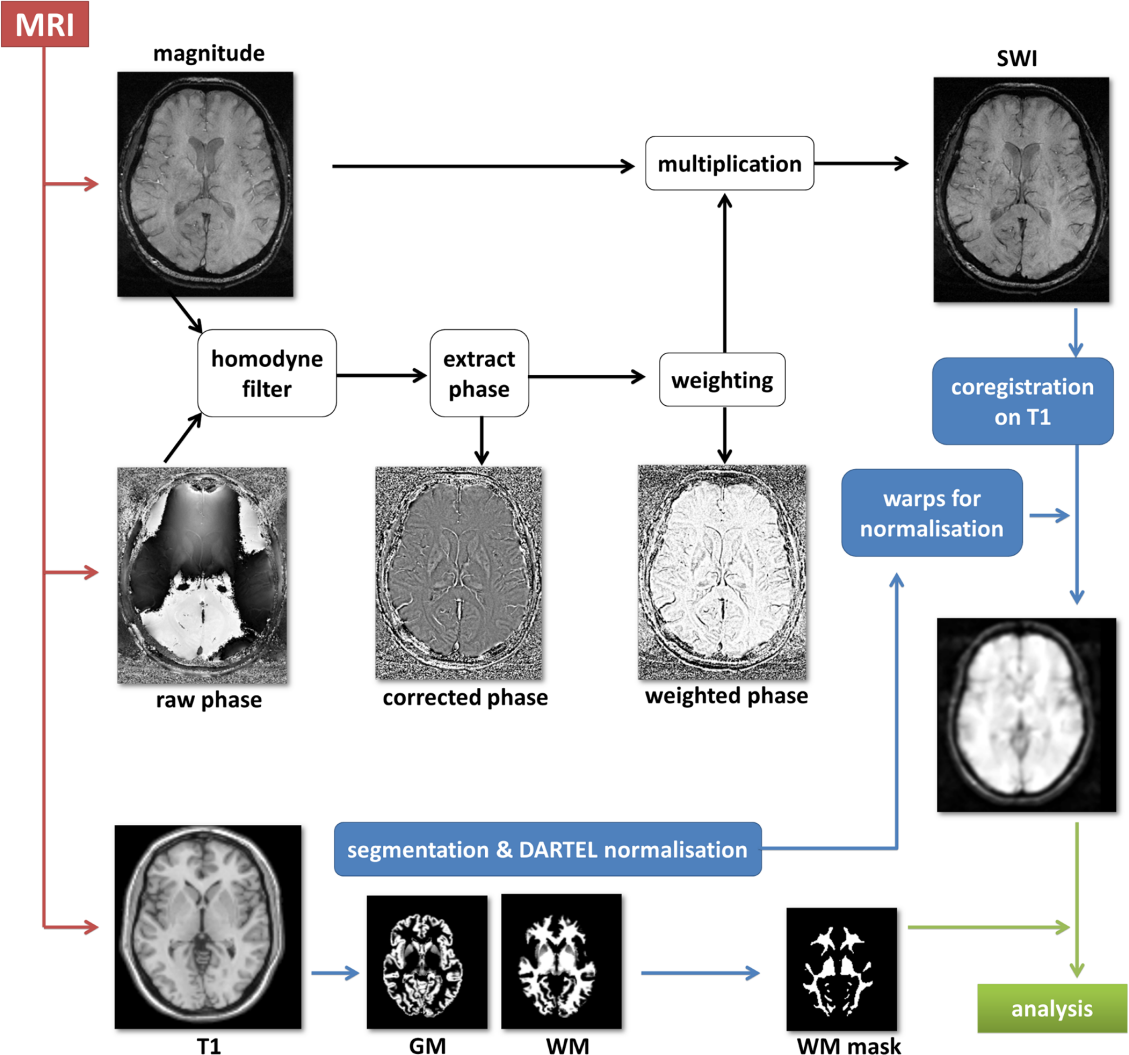
Scheme of the data SWI processing pipeline.

All scans were obtained on a 1.5 Tesla Siemens Sonata scanner using a single-channel head coil. All participants were comfortably placed with their heads immobilized with cushions during scanning. SWI data were acquired with a 3D, rf-spoiled, single-echo gradient echo (GRE) sequence with first order flow-compensation in all three spatial directions. The following sequence parameters were used: 40 ms echo time, 57 ms repetition time, 20° flip angle, 448 x 352 data matrix, 0.54 x 0.54 x 2 mm^3^ voxel size, 50 Hz/px bandwidth. SWI images were calculated by combining magnitude and phase data according to Fig 1 [12]. Homodyne filtering was performed to correct raw phase images for phase wraps and for background phase contributions [13, 10]. To this end, the complex-valued GRE data were divided in the image domain with a complex-valued, low-pass filtered copy of the same data. Taking the arc tangent of these modified data yields corrected phase images, ϕ_c_. Low-pass filtering was carried out by slice-wise multiplication of the original GRE data with a 2D Hanning window in the Fourier domain using a width of 25% of the data matrix size [14]. Since a MRI scanner with left-handed static magnetic field orientation was used in our study, the corrected phase images, ϕ_c_, were converted into a normalised, weighted phase mask, ϕ_w_, in the following way [12]:

$$\varphi_{w}=f(\varphi_{c})=\left\{ \begin{array}{l} \;(1-\frac{\varphi_{c}}{\pi })\\ 1 \end{array}\right.\begin{array}{c} 4 \end{array}\begin{array}{c} \;,if\varphi_{c}\geq 0\\ \;,if\varphi_{c}<0 \end{array}$$

This phase mask was used to enhance image contrast due to paramagnetic structures such as iron-laden tissue and venous vessels by multiplying it with the corresponding magnitude image to obtain the so called susceptibility weighted images (SWI) [8, 15]

Image preparation for subsequent analysis was performed using Dcm2Nii (MRIcroN, http://www.mccauslandcenter.sc.edu/mricro/mricron/dcm2nii.html) for conversion into the NifTi format. Data were then further preprocessed by using MATLAB R2009b (TheMathworks, Natick, USA) as mathematical framework, SPM8 (Wellcome Trust Centre for Neuroimaging, UCL, London, UK) and the VBM8 toolbox (http://dbm.neuro.uni-jena.de/vbm/download/). SWI and their corresponding 3D T1-weighted images (acquired in the same MRI session) were reoriented into oblique para-axial slices aligned parallel to the anterior-posterior commissural axis with the origin set to the anterior commissure. SWI images were co-registered onto the corresponding T1 images. T1 images were normalised into MNI space using DARTEL. The warps from this normalisation procedure were then used to normalise SWI data into MNI space. A study specific white matter mask was created from all T1 images by using the VBM8 toolbox. This mask was later used as the explicit mask in the analyses. Finally, SW images were smoothed with a 4 mm FWHM Gaussian kernel.

All image data are available via the NeuroImaging Society in ALS (NISALS) which is an interactive platform for research centers specializing in ALS Neuroimaging research (www.nisals.org).

### Statistical analysis

Processed SW images were analyzed with a 90% inclusive probability white matter mask derived from the corresponding VBM sections to reduce the search volume to white matter compartments. Group comparison between ALS patients and healthy controls for the whole brain was performed with SPM8 using the model *'one-way ANOVA*' with age and sex as nuisance variate. After threshold-free cluster enhancement (TFCE) [16] the resulting statistical parametric maps were thresholded at *P* < 0.05 and corrected for multiple comparisons using the family wise error method (FWE). Additionally, these observed significant clusters from group comparison were correlated with the disease severity (ALSFRS-R) by using the SPM8 model *'multiple regression'*. These resulting statistical parametric maps were thresholded at *P* < 0.05 and corrected for multiple comparisons using FWE. Anatomical location was determined with the Jülich White Matter atlas implemented in FSL (http://fsl.fmrib.ox.ac.uk). Finally, the statistical parametric maps were overlaid on T1 slices using MRIcroN.

## Results

**Fig 2:**
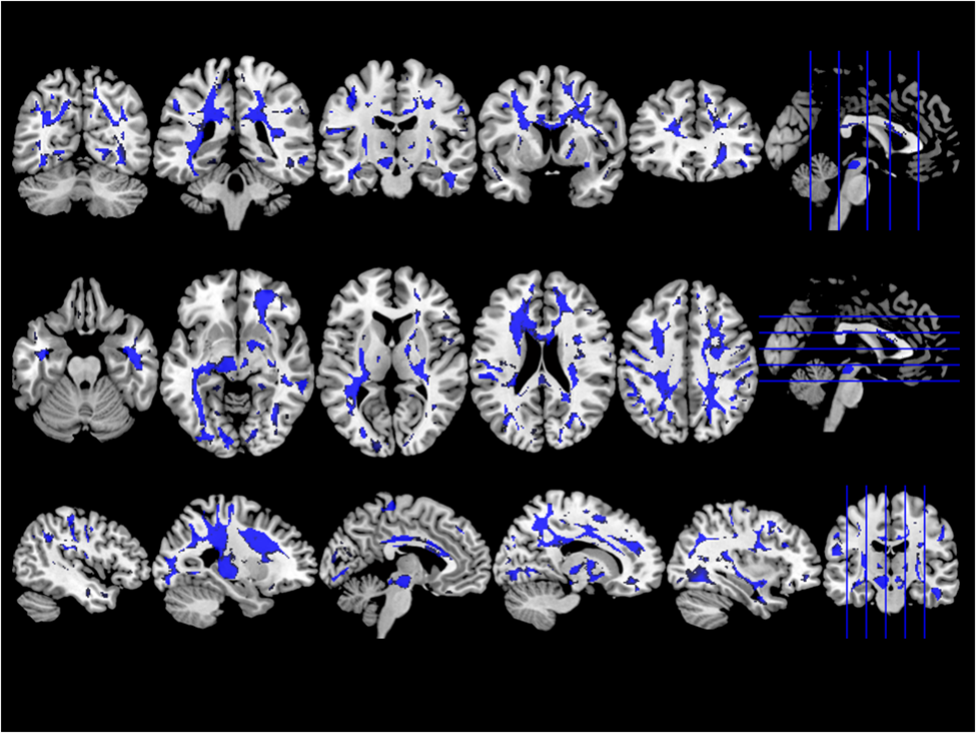
Group comparison of SWI images between ALS patients and healthy controls. ALS patients showed lower SWI signal in deep white matter tracts, including corpus callosum, corticospinal and superior longitudinal fascicle most prominent in its frontal parts. The statistical parametric maps are displayed at a threshold *P* < 0.05 and corrected for multiple comparisons using FWE.

Patients with ALS showed significantly lower SWI signal intensity in the deep white matter tracts along the whole CST ranging from the pre- and postcentral gyri throughout the posterior limb of the internal capsule until the brainstem. Regional differences between patients with ALS and healthy controls were also observed in the superior and inferior longitudinal fasciculus, anterior thalamic radiation, cingulum, the splenium and body of the corpus callosum, as well as in the inferior fronto-occipital fasciculus (Fig 2).

Disease severity (ALSFRS-R) correlated negatively with the SWI changes in the body of the corpus callosum and the corticospinal tract (mainly on the right side). There were no significant differences in SWI between male and female patients.

## Discussion

This study employed SWI to detect white matter pathology in patients with ALS. We found widespread white matter alterations in the brains of patients. These changes were not restricted to the CST and clearly extended to the frontal, temporal, parietal and occipital lobes. Thus, SWI can detect the imaging hallmarks of white matter pathology in patients with ALS.

The observed signal alterations are in line with reported microstructural white matter changes detected in plenty of studies using diffusion tensor imaging (DTI), which is sensitive to alterations in the degree (apparent diffusivity, ADC) and directedness (fractional anisotropy, FA) of proton movements. DTI is a promising biomarker in ALS and has been shown to reveal widespread white matter abnormalities that are most prominent along the CST and in the subcentral white matter, extending into the frontal, temporal and cerebellar regions [7]. Our study provides further evidence that involvement of the corpus callosum is a consistent feature of ALS [17].

### Widespread white matter damage in ALS

We observed structural changes in the motor and extramotor areas. Involvement of the CST could be demonstrated below the motor cortex, in the posterior limb of the internal capsule and brainstem. This is in line with the hallmark degeneration observed in neuropathological studies of ALS brains [6]. In agreement with the description of asymmetry in ALS pathology [18], we found a correlation with disease severity mainly at the right CST. We also observed frontal lobe white matter alterations. Frontal executive dysfunction is common in patients with ALS, and has been recently described in genetic and neuropathological studies, underlining overlap between ALS and frontotemporal dementia (FTD) [19]. Therefore, the observed involvement of the frontal, temporal and parietal lobes is in line with the known spectrum of cognitive, genetic (e.g. C9ORF72) and pathological overlap between ALS and FTD. However, we were unable to validate this in our cohort–although none of our patients exhibited frank dementia–because no neurogenetic testing or extensive neuropsychological assessments had been performed. In the context of FTD and ALS, it is interesting that we observed abnormalities in the cingulate cortex and adjacent white matter tracts in the corpus callosum, which is a hallmark degenerative characteristic of ALS and FTD [20]. The cingulate cortex is highly connected with other paralimbic and limbic structures; areas of heteromodal association cortex in the frontal, temporal and parietal lobes; dorsolateral prefrontal cortex and subcortical regions, including the thalamus and striatum [21] and thus plays an important role in cognition and attention regulation. It exhibits abnormal structure and function in neurodegenerative diseases, such as amyloid deposition in patients with Alzheimer´s disease [21]. Cortical thinning has been recently described in the posterior cingulate cortex of patients with ALS–FTD [22].

Furthermore, we observed white matter damage in the occipital lobe, in particular beneath the middle occipital gyrus, lingual gyrus, precuneus and cuneus. Involvement of the occipital lobe has been previously demonstrated in patients with ALS by cortical thinning of the medial and lateral occipital areas bilaterally [23]. The middle occipital gyrus is relevant for face and tool processing [24], and the involvement of white matter below this region in our study provides further evidence of generally altered processing of visual and emotional information in patients with ALS [25]. The occipital lobe has fronto-occipital and temporo-occipital connections, and white matter damage in these tracts was found to be correlated with performance in tests assessing attention and executive functions [26].

### Neuropathological basis of the observed signal alterations

The SWI technique utilizes magnitude and phase images to generate a specific combined contrast that indicates the presence of paramagnetic or diamagnetic tissue changes. In clinical settings, the blood oxygen level-dependent components are typically used to, e.g., trace venous vessels and/or hemorrhages [3, 27, 28].

Although the resulting susceptibility contrast is strongly linked to the concentration of paramagnetic storage iron, it is also influenced by the integrity of diamagnetic myelin, which in turn is highly dependent on an adequate iron metabolism. Disturbances in iron metabolism and myelin damage can occur at the same time in ALS and both largely influence susceptibility. Therefore, in a statistical approach focusing on white matter tracts, SWI may help to assess myelin integrity. However, SWI–as almost all MRI techniques–only delivers an aggregated marker of susceptibility features of all molecules in the scanned volume. Because enhanced iron levels are considered to participate in oxidative stress and neuronal death in patients with ALS [29], myelin damage could be a reason for the following observed signal alterations [30]: (i) with a positive phase mask used to generate the SW images, hypointense signals are indicative of less diamagnetic tissue being locally present (e.g. due to a breakdown of diamagnetic myelin) and (ii) increased magnitude signal (hyperintensity) may be due to increased T_2_*, which in turn is also indicative of a breakdown of diamagnetic myelin and cannot be explained by accumulation of paramagnetic iron [31]. The deposition of iron in the precentral gyrus of patients with ALS has been discussed for many years [32, 33]. In a more recent study including 15 patients with ALS and 15 age- and gender-matched controls, assessing the iron levels by T_2_* mapping and DTI measurements that focused on the CST and on deep grey matter structures, patients with ALS showed decreased T_2_* signals, which is indicative of iron deposition along the CST. Concerning disease severity (ALSFRS-R), no significant cluster differences for R_2_* (= 1/T_2_*) or water mobility (FA, mean diffusivity) were found in the major white matter tracts [34]. However, it should be noted that T_2_* changes do not necessarily reflect disturbances in the iron levels, but that microstructural tissue changes can also change T_2_* relaxation. Since our study design was not restricted to specific brain regions we could therefore demonstrate that signal alterations caused by iron and myelin disturbances clearly extend beyond the motor system reaching into the frontal, temporal, limbic and occipital regions, and thus support the current view of ALS as a multisystem disorder.

In summary, we believe that SWI is a promising biomarker for ALS. Its usefulness as diagnostic and monitoring biomarker needs to be evaluated, however, in longitudinal study designs with larger cohorts.

### Methodological issues and limitations

There are several limitations of our study due to its primary aim of demonstrating the utility of using SWI in patients suffering from ALS. Our cohort of patients was not analysed with respect to their onset type because the bulbar-onset group was too small to obtain valid results. We also did not undertake extensive neuropsychological or genetic testing, which would be helpful to estimate the influence of frontotemporal disturbances. Although SWI is also capable to detect grey matter changes, this study was restricted to the analysis of white matter for several reasons. The inter-individual differences in the comparison of human brains are smaller in white matter than in grey matter, such as the motor cortex. Additionally white matter changes are of great interest in terms of diagnosing and monitoring biomarker in ALS. Due to the aim of the study–showing proof of principle of SWI in ALS–we did not perform a comparison to other MRI techniques, such as DTI in the same cohort. This would be useful for further studies in larger cohorts of patients with ALS.
